# The Thermal Stability of the Collagen Triple Helix Is Tuned According to the Environmental Temperature

**DOI:** 10.3390/ijms23042040

**Published:** 2022-02-12

**Authors:** Kazunori K. Fujii, Yuki Taga, Yusuke K. Takagi, Ryo Masuda, Shunji Hattori, Takaki Koide

**Affiliations:** 1 Department of Chemistry and Biochemistry, School of Advanced Science and Engineering, Waseda University, Shinjuku, Tokyo 169-8555, Japan; k.fujii@ruri.waseda.jp (K.K.F.); takagi.yt@moegi.waseda.jp (Y.K.T.); 2 Nippi Research Institute of Biomatrix, 520-11 Kuwabara, Toride 302-0017, Japan; y-taga@nippi-inc.co.jp (Y.T.); shunhatt@nippi-inc.co.jp (S.H.); 3 Waseda Research Institute for Science and Engineering, Waseda University, Shinjuku, Tokyo 169-8555, Japan; r.masuda@aoni.waseda.jp

**Keywords:** collagen, triple helix, environmental temperature, post-translational modification, thermal stability

## Abstract

Triple helix formation of procollagen occurs in the endoplasmic reticulum (ER) where the single-stranded α-chains of procollagen undergo extensive post-translational modifications. The modifications include prolyl 4- and 3-hydroxylations, lysyl hydroxylation, and following glycosylations. The modifications, especially prolyl 4-hydroxylation, enhance the thermal stability of the procollagen triple helix. Procollagen molecules are transported to the Golgi and secreted from the cell, after the triple helix is formed in the ER. In this study, we investigated the relationship between the thermal stability of the collagen triple helix and environmental temperature. We analyzed the number of collagen post-translational modifications and thermal melting temperature and α-chain composition of secreted type I collagen in zebrafish embryonic fibroblasts (ZF4) cultured at various temperatures (18, 23, 28, and 33 °C). The results revealed that thermal stability and other properties of collagen were almost constant when ZF4 cells were cultured below 28 °C. By contrast, at a higher temperature (33 °C), an increase in the number of post-translational modifications and a change in α-chain composition of type I collagen were observed; hence, the collagen acquired higher thermal stability. The results indicate that the thermal stability of collagen could be autonomously tuned according to the environmental temperature in poikilotherms.

## 1. Introduction

Collagen is the most abundant protein in vertebrates. As the major component of the extracellular matrix, it plays roles not only in maintaining tissue structure but also in regulating cellular functions. A total of 28 types of collagen have been identified in humans [1]. Of these, type I collagen, the most abundant fibrillar collagen, has been well studied. Type I collagen has a triple-helical structure consisting of three polypeptide chains, called α-chains, containing > 1000 amino-acid residues. Each α-chain contains more than 330 repeats of the Gly-X-Y triplet, with approximately one-third of positions X and Y occupied by proline (Pro) and 4-hydroxyproline (4-Hyp) residues, respectively.

The major post-translational modification of collagen, 4-hydroxylation of Pro, contributes significantly to the stabilization of the triple helix [2]. The number of 4-Hyp residues at position Y is positively correlated with the thermal denaturation temperature of the collagen triple helix [3,4,5]. Other collagen-specific post-translationally modified amino-acid residues include 3-Hyp at position X and hydroxylysine (Hyl) at position Y. Hyl residues are further glycosylated to form galactosyl-hydroxylysine or glucosyl-galactosyl-hydroxylysine residues. 3-Hyp was reported to enhance the thermal stability of the collagen triple helix [6]. However, no consensus on the effects of the lysine (Lys) side chain modifications exists [7,8].

The thermal stability of collagen in carp’s scales collected in summer was 1.8 °C higher than that obtained in winter [9]. Thus, the thermal stability of collagen in organisms grown at different environmental temperatures may vary within the same species or individuals.

The folding and post-translational modification of procollagen, a collagen precursor, occurs in the lumen of the endoplasmic reticulum (ER). The three pro α-chains first associate at the C-propeptide domain and form a triple helix from C- to N-terminus [10,11]. Post-translational modifications are catalyzed by enzymes that recognize single-stranded polypeptides as their substrates [12]. Procollagen molecules with complete triple-helical domains are transported to the Golgi apparatus and then secreted into the extracellular space. Procollagen molecules that fail to form the triple helix are kept in or returned to the ER, and subsequently, degraded through autophagy [13].

HSP47, a procollagen-specific molecular chaperone in the ER of vertebrates, is constitutively expressed but is the only heat-shock protein found in the secretory pathway. The chaperone recognizes the triple-helical portions of procollagen in the lumen of the ER, thereby inhibiting the aggregation of procollagen [14] and/or stabilizing the triple-helical structure [15,16]. HSP47 dissociates in a pH-dependent manner in the *cis*-Golgi or ER-Golgi intermediate compartment and is then retrieved to the ER. The *hsp47*-knockout mice showed an embryonic lethal phenotype due to extensive collagen abnormalities, indicating that HSP47 is an essential chaperone at least in mammals [17]. In addition, various abnormalities have been observed in fibroblasts established from *hsp47*-knockout mice [14,17,18] and in cells obtained from dachshunds [19] and humans [20] with recessive mutations in the *hsp47* gene. They show accumulation of procollagen in the ER and excessive post-translational modifications of collagen.

Previously, we reported an increase in the number of post-translational modifications as well as in the thermal denaturation temperature and ratio of the (α1)_3_ homotrimer of type I collagen secreted by *hsp47*-knockout mouse fibroblasts [18]. Furthermore, the abnormalities observed at 37 °C were rescued by incubating the cells at a lower temperature of 33 °C. These results indicated the essential function of HSP47 in stabilizing the triple-helical portions of procollagen, allowing for procollagen folding, which is not possible by the protein alone. Moreover, the properties of collagen, such as the number of post-translational modifications and thermal denaturation temperature of the triple helix, are affected by environmental temperature. However, there was little difference in the properties of collagen secreted from normal mouse fibroblasts cultured at 37 or 33 °C. In this study, by using poikilotherm cells, we tested the hypothesis that procollagen is post-translationally modified in the ER lumen until it forms a triple helix and acquires enough thermal stability to maintain the structure at the environmental temperature. Zebrafish are known to be tolerant to a wide range of temperatures (10–40 °C) [21,22]. Furthermore, zebrafish type I collagen has an α3-chain in addition to α1- and α2-chains found in mammals. The α3-chain possesses high homology to α1-chain and is proposed to be incorporated as a surrogate of the α1-chain in zebrafish type I collagen [23]. In this study, zebrafish fibroblasts were cultured at temperatures between 18 and 33 °C, and the thermal stability and post-translational modifications of secreted collagen, as well as the α-chain compositions of type I collagen, were analyzed.

## 2. Results

### 2.1. Western Blot Analysis of Proteins Expressed by ZF4 Cells Cultured at Different Temperatures

**Figure 1:**
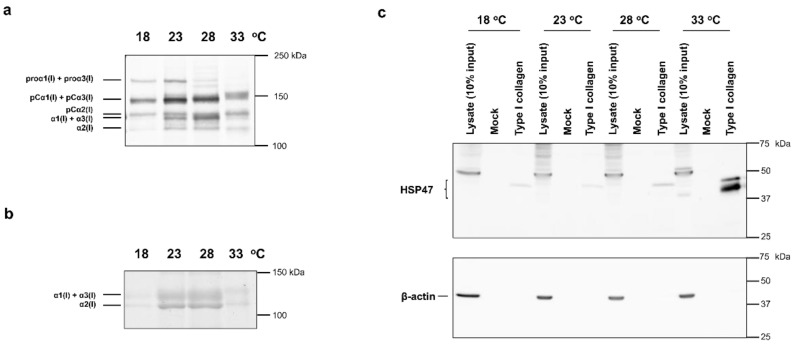
Analysis of extracellular collagen and intracellular HSP47 proteins in ZF4 cells cultured at different temperatures. (**a**) The loaded amount of SDS samples prepared from culture media was normalized to the protein amount of the cell layer. SDS samples are loaded onto SDS-PAGE gel (5%) under reducing conditions. After transfer to a nitrocellulose membrane, collagen polypeptides were detected by using biotinylated soCMP6-7(Glu)2. (**b**) The culture media of ZF4 cells was treated with pepsin at 4 °C, and the triple-helical region of collagen was purified through salt precipitation. SDS-PAGE (5%) was performed under nonreducing conditions. Proteins were visualized with CBB. (**c**) HSP47 was pulled down from the lysate of ZF4 cells using type I collagen-coupled beads or mock-coupled beads. The SDS samples were subjected to SDS-PAGE (10%) under nonreducing conditions, and western blotting was performed using an antibody against HSP47 (top). The same membranes were reprobed with the anti-β-actin antibody (bottom).

In this study, zebrafish embryonic fibroblasts (ZF4) were used. Although ZF4 cells are cultured at approximately 28 °C, the cells could proliferate at 18, 23, 28, and 33 °C (data not shown). ZF4 cells were first cultured to confluence at 28 °C, and then at 18, 23, 28, and 33 °C for 3 days. Then, the media were collected, and western blotting using biotinylated denatured collagen-binding peptide (soCMP6-7(Glu)2) [24] was performed to compare the amount of secreted collagen normalized to protein content in the cell layer (Figure 1a). Because the molecular sizes of α1(I) and α1(I) with the unprocessed C-terminal propeptide (pCα1(I)) are almost equal to those of α3(I) and α3(I) with unprocessed C-terminal propeptide (pCα3(I)), respectively, their bands could not be resolved by SDS-PAGE. The amount of secreted collagen was relatively small at low (18 °C) and high (33 °C) temperatures compared with the medium (23 and 28 °C) temperatures. In particular, the band density of pCα1(I) + pCα3(I), α1(I) + α3(I), and α2(I)-chains in the lanes of the 23 and 28 °C samples were higher than those of the 18 and 33 °C samples. In addition, the electrophoretic mobilities of pCα1(I) + pCα3(I), pCα2(I), and α1(I) + α3(I)-chains were slightly lower in the 33 °C sample than in the other samples, suggesting that the molecular size of these polypeptide chains increased at 33 °C.

We next investigated whether the increase in the molecular size of collagen was attributed to the increase in the molecular size of the triple-helical region. We analyzed collagen samples prepared by pepsin treatment at 4 °C, followed by salt precipitation of culture supernatant. The collagen samples were separated by SDS-PAGE, and the protein bands were visualized by Coomassie Brilliant Blue R-250 (CBB) staining. The electrophoretic mobilities of α1(I) + α3(I) and α2(I)-chains secreted at 33 °C decreased compared with those secreted at other culture temperatures (Figure 1b). These results suggest that excessive post-translational modifications occur in the triple-helical region of collagen at 33 °C. Considering the difference in band mobilities, we hypothesized an increase in lysyl glycosylation at 33 °C. Furthermore, a comparison of α1(I) + α3(I) and α2(I) band intensities in each sample revealed that the ratio of α1(I) + α3(I) to α2(I) was relatively higher in the sample at 33 °C than at other temperatures.

Because the procollagen-specific chaperone HSP47 is heat-inducible, we examined whether HSP47 content changes according to the culture temperature. ZF4 cells cultured for 3 days at 18, 23, 28, and 33 °C were lysed in lysis buffer containing 1% Nonidet P-40 (NP-40). HSP47 was concentrated from the lysates using type I collagen-immobilized beads. The amount of HSP47 in the samples was compared by western blotting (Figure 1c). β-Actin was used as a loading control. Although the anti-HSP47 (anti-SERPINH1) polyclonal antibodies could not detect zebrafish HSP47 sensitively in cell lysates, the HSP47 bands were detectable after the protein was concentrated using collagen-immobilized beads. Bands at approximately 50 kDa may be nonspecific because they were observed only in cell lysate samples and did not exhibit collagen binding. The amount of HSP47 was almost constant from 18 to 28 °C, whereas it significantly increased at 33 °C. Previous microarray analysis of zebrafish gene expression showed that HSP47 gene expression was almost the same below 28 °C but increased more than 10-fold at a higher temperature (34 °C) [25], which is consistent with the results of this study. Two distinct bands of HSP47 were detected in the sample for the 33 °C culture (Figure 1c). In addition to HSP47 (*serpinh1b*), zebrafish have HSP47-like (*serpinh1a*), which may have been detected as different bands. However, both HSP47 and HSP47-like have two glycosylation sites; therefore, the two bands may represent differences in glycosylation [26].

### 2.2. Quantification of Total Post-Translational Modifications of Collagen

**Figure 2:**
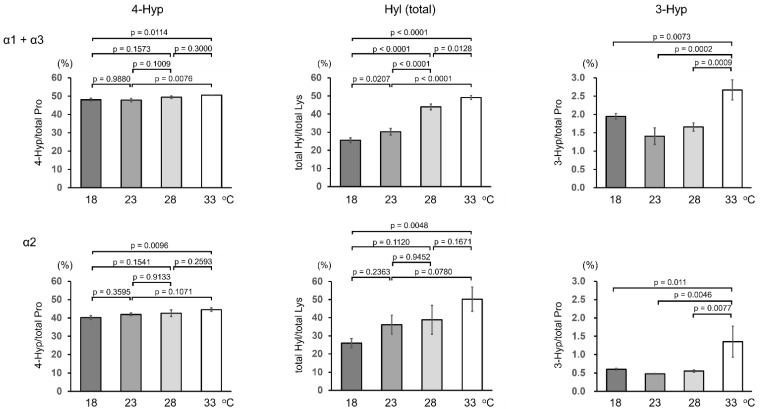
Quantification of total post-translational modifications in type I collagen secreted from ZF4 cells. Culture media of ZF4 cells grown at 4 °C was pepsin-treated, and collagen was purified through salt precipitation. Purified collagen was subjected to SDS-PAGE and transferred to a PVDF membrane. Then, α1(I) + α3(I) and α2(I) bands were excised and subjected to acid hydrolysis with SI-collagen. Pro, Lys, 3-Hyp, 4-Hyp, and total Hyl (Hyl + glycosylated Hyl) were quantified using LC-MS. Values for 4-Hyp (4-Hyp/(Pro + 4-Hyp + 3-Hyp)), total Hyl (total Hyl/(Lys + total Hyl)), and 3-Hyp (3-Hyp/(Pro + 4-Hyp + 3-Hyp)) ratios were calculated. Values are means ± SD (*n* = 3). Differences of means were assessed by one-way ANOVA followed by a Tukey’s post-hoc test.

The molecular size of the triple-helical region of collagen secreted by ZF4 cells at 33 °C increased (Figure 1b), suggesting an increase in the number of post-translational modifications. We performed amino-acid analysis of acid-hydrolyzed samples to compare the degree of post-translational modification occurring at different culture temperatures. ZF4 cells were cultured for 3 days at 18, 23, 28, or 33 °C, and the triple-helical region of collagen was purified from the culture media through pepsin digestion and salt precipitation. The collagen samples were separated using SDS-PAGE, followed by transfer to a polyvinylidene fluoride (PVDF) membrane. Bands of α1(I) + α3(I) and α2(I) were excised, and stable isotope-labeled human collagen (SI-collagen) was added to the excised bands as an internal standard for LC-MS analysis of Pro, 3-Hyp, 4-Hyp, Lys, and total Hyl (Hyl and glycosylated Hyl) generated after acid hydrolysis [27]. The quantitative results, expressed as residues per 1000 residues, are shown in Table S1. From the results obtained, 4-Hyp ratio (4-Hyp/(Pro + 4-Hyp + 3-Hyp)), 3-Hyp ratio (3-Hyp/(Pro + 4-Hyp + 3-Hyp)), and total Hyl ratio (total Hyl/(Lys + total Hyl)) were estimated (Figure 2).

The 4-Hyp and total Hyl ratios increased in a temperature-dependent manner in both α1(I) + α3(I) and α2(I). The ratio of Pro residues at position Y to all Pro residues in the triple-helical region of zebrafish collagen is 50% for α1(I) + α3(I) and 43% for α2(I) (Table S2a). The 4-Hyp ratio, even in the collagen secreted from ZF4 at 18 °C, was 48% in α1(I) + α3(I) and 40% in α2(I). Because almost all Pro residues at position Y were converted to 4-Hyp, few Pro residues to remained be 4-hydroxylated, resulting in the relatively small increase in the 4-Hyp ratio with increasing temperature (Figure 2, left). Lys residues at position Y, which can be modified to Hyl, account for 66% and 63% of Lys in α1(I) and α3(I), respectively, and 71% in α2(I) (Table S2b). In collagen secreted from ZF4 cells at 18 °C, only 26% of Lys in both α1(I) + α3(I) and α2(I) was converted to Hyl, indicating that many sites remained unmodified. As the environmental temperature increased, the Hyl ratio increased remarkably, reaching 49% in α1(I) + α3(I) and 50% in α2(I) at 33 °C (Figure 2, middle). Acid-labile glycosylated Hyl could not be analyzed here. However, given the increasing degrees of the analyzed modifications at 33 °C and slight mass shift upon hydroxylation of Pro and Lys (+16 Da) residues, the decrease in electrophoretic mobility of collagen at the higher temperature was strongly suggestive (Figure 1b) of the increase in Hyl glycosylation. By contrast, the number of 3-Hyp residues markedly increased only in collagen secreted from ZF4 cells at 33 °C (Figure 2, right). These results indicate that the increase in environmental temperature increases the number of post-translational modifications, but the temperature sensitivity was different according to modification types.

### 2.3. Quantification of Site-Specific 3-Hyp Modification

Focusing on prolyl 3-hydroxylation, a relatively rare post-translational modification in collagen, we further analyzed the effect of culture temperature on prolyl 3-hydroxylation at specific Pro residues of each α-chain in type I collagen. Although information on the positions of 3-hydroxylation in fish collagen is absent, the known sites (Pro^707^, Pro^716^, Pro^719^, and Pro^986^ at position X of Gly-X-4-Hyp) in mammalian collagen for the modification are mostly conserved in zebrafish (Figure S1) [28].

**Figure 3:**
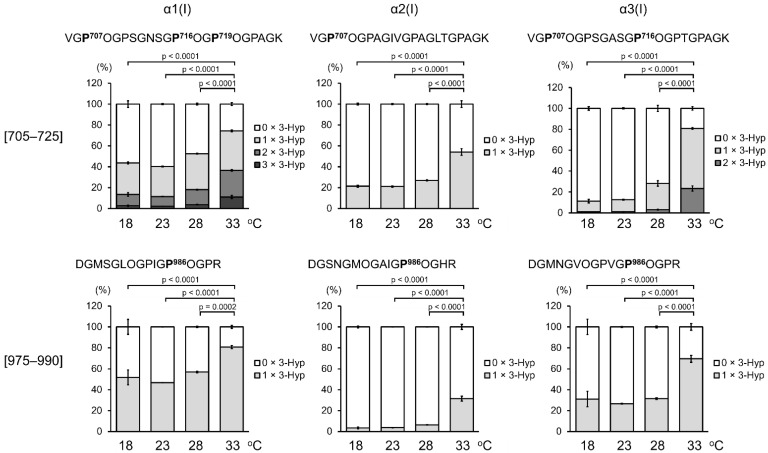
Quantification of site-specific 3-Hyp in type I collagen secreted from ZF4 cells. After pepsin treatment of culture media of ZF4 cells at 4 °C, collagen was purified through salt precipitation. Purified collagen was heat denatured and trypsin digested. LC-MS analysis was performed to quantify 3-Hyp at specific sites (indicated as boldface letters in the sequences). O indicates 4-Hyp. Values are means ± SD (*n* = 3). Differences of means in the 0 × 3-Hyp (unmodified Pro) were assessed by one-way ANOVA followed by Tukey’s post hoc test.

ZF4 cells were cultured for 3 days at different temperatures. Collagen samples were prepared and subjected to trypsin digestion to estimate the relative abundance of 3-Hyp at the specific sites using LC-MS (Figure 3).

Reportedly, in types I and II collagen of teleosts, including zebrafish, Pro is not hydroxylated at the 3-hydroxylation sites known in mammals [29]. In this study, LC-MS analysis revealed a +16 Da mass shift of Pro residues at the known 3-hydroxylation sites in α1(I), α2(I), and α3(I) of zebrafish. These findings strongly suggest the presence of 3-Hyp at these sites, which is consistent with results from a study on rat collagen [28]. Furthermore, although 3-hydroxylation of α2(I) Pro^986^ has not been reported in mammals, 3-hydroxylation of this site was detected in ZF4 cells (Figure S1). In collagen secreted from ZF4 cells at 18, 23, and 28 °C, no marked difference in the number of 3-Hyp residues at any analyzed peptide fragment was observed; however, 3-Hyp number markedly increased only in collagen secreted from ZF4 cells at 33 °C (Figure 3). These results are consistent with changes in the number of 3-Hyp residues at different temperatures (Figure 2).

### 2.4. α-Chain Ratios of Type I Collagen Secreted from ZF4 Cells

**Figure 4:**
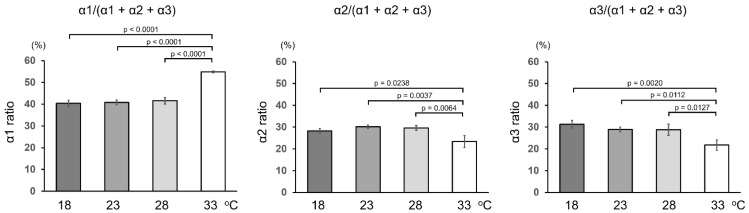
Quantification of type I collagen α-chains secreted from ZF4 cells. Pepsinized collagen was purified from the culture media through salt precipitation. The samples were digested with trypsin after adding stable isotope-labeled synthetic peptides and heat denatured. The amount of each α-chain of type I collagen was determined through LC-MS analysis of marker peptides. Values are means ± SD (*n* = 3). Differences of means were assessed by one-way ANOVA followed by Tukey’s post hoc test.

Because the number of each α-chain in secreted type I collagen changed according to the culture temperature (Figure 1a,b), we quantified the relative number of α-chains. Collagen samples purified from the culture media of ZF4 cells were digested with trypsin and the generated marker peptides of each α-chain were analyzed using LC-MS (Figure 4 and Tables S3 and S4). We used ^15^N-enriched synthetic peptides (Figure S2 and Table S5) as internal standards for MS analysis. The ratio of each α-chain to total α-chains was almost constant below 28 °C. However, at 33 °C, the α1 ratio increased (from 40–42% to 55%) and decreased both α2 and α3 ratios (from 28–31% to 22–23%). This result indicates that the proportion of α-chains in type I collagen secreted from ZF4 cells depends on culture temperature, and dramatic changes occurred around 33 °C.

The positions of cysteine residues in the C-propeptide domains of α-chains were reported to determine the combinations of α-chains involved in triple helix-formation [30]. Trimers with the chain-arrangements of (α1)_3_, [(α1)_2_α3], [(α1)_2_α2], and [α1α2α3] are possible for zebrafish type I collagen. Using data shown in Figure 4 and Table S4, we simulated the ratios of these trimers secreted from ZF4 cells at each temperature. Details of the calculations are described in Section 4.8. Because quantification data were insufficient to calculate the actual ratios of the trimers, the calculation was performed assuming variables x, y, z, and w (%), as the ratios of [(α1)_2_α3] at 18, 23, 28, and 33 °C, respectively. As shown in Table 1, the ratio of [α1α2α3] to total trimer was at least 75.3–78.5% at 18–28 °C, but at most 65.2% at 33 °C, indicating that the [α1α2α3] ratio decreased at 33 °C compared with other temperatures. A wide range of values due to the influence of variables made a precise comparison of other trimer ratios among different temperatures challenging. Instead, of comparing individual ratios of trimers, the sum of the ratios of two trimers was compared. The sum of ratios of (α1)_3_ and [(α1)_2_α3] and that of ratios of (α1)_3_ and [(α1)_2_α2] were estimated to be highest at 33 °C (Table 1). The sum ratio values of (α1)_3_ and [(α1)_2_α3] were calculated to be 15.1% at 18 °C, 9.4% at 23 °C, 11.1% at 28 °C, and 29.9% at 33 °C. Those of (α1)_3_ and [(α1)_2_α2] were estimated to be 6.1% at 18 °C, 13.1% at 23 °C, 13.6% at 28 °C, and 34.8% at 33 °C.

### 2.5. Thermal Stability of Collagen Triple-Helical Structure Secreted from ZF4 Cells

**Figure 5:**
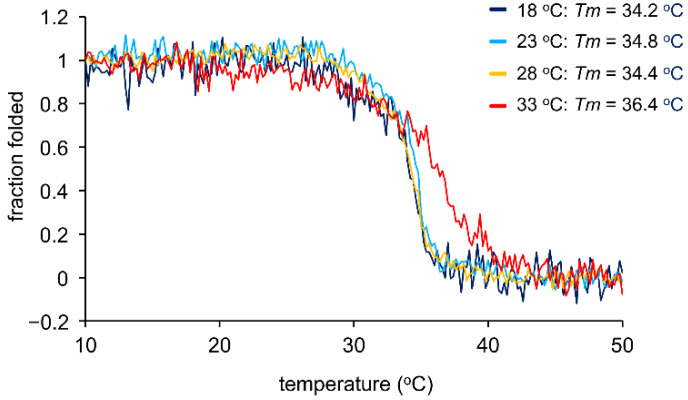
Thermal melting curves for collagen triple helix obtained by CD spectrometry. ZF4 cells were cultured for 3 days at the temperatures indicated. After the culture media were treated with pepsin at 4 °C, collagen was isolated by salt precipitation. CD signal was measured at 221 nm with increasing temperature (0.25 °C/min). The melting temperature (*Tm*) was set to the temperature at which the fraction folded was 0.5.

We compared the thermal stability of the triple-helical structure of type I collagen secreted from ZF4 cells cultured at different temperatures. The triple helix collagen samples were similarly prepared, and polypeptide chain conformation was analyzed by circular dichroism (CD) spectrometry. Thermal melting curves were obtained by plotting fraction folded, calculated by measuring ellipticity values at 221 nm, with increasing temperature (Figure 5). The melting temperatures of the collagen triple helix were estimated to be 34.2, 34.8, 34.4, and 36.4 °C for the 18, 23, 28, and 33 °C samples, respectively. This result indicates that the thermal stability of type I collagen is almost constant below 28 °C and is markedly enhanced at a higher temperature (33 °C). This increase in thermal stability may be caused by an increase in post-translational modification and/or a change in the α-chain composition of type I collagen, which was observed in the experiments described above.

To investigate the effect of the α-chain arrangements on the thermal stability of the triple helix, we performed computational simulation and compared the relative thermal stability of the triple helices of zebrafish type I collagen molecules with different chain arrangements. We used SCEPTTr, an empirical algorithm that predicts the melting temperature of the triple-helical structure of collagen-model peptides [31]. In the collagen triple helix, three polypeptide chains intertwine with one another, with one-residue staggers along the helical axis. Therefore, in a heterotrimer consisting of two A-chains and one B-chain, the B-chain occupies either the leading (BAA), middle (ABA), or trailing (AAB) position, resulting in three isomers. There are six isomers in a heterotrimer consisting of one A-chain, one B-chain, and one C-chain, accordingly. Considering that only (α1)_3_, [(α1)_2_α2], [(α1)_2_α3], and [α1α2α3] are suggested to form zebrafish type I collagen, we predicted 13 isoforms, including chain-staggering isomers. Of these, we excluded three chain-staggering isoforms having α2-chain as the middle strand, because α1α2α1 is unlikely to form in mammals [32,33], and zebrafish α3-chain can be placed as a surrogate for the α1-chain, judging by their homology [23]. Taken together, we hypothesized that ten different type I collagen molecules can form in zebrafish (Table 2).

Each isoform’s virtual relative thermal stability was calculated as the average of the predicted melting temperatures for 334 collagen-derived peptide fragments of 45 amino-acid residues flanked by (Pro-Hyp-Gly)_5_ at both N- and C-terminus. All Pro residues at position Y were substituted with 4-Hyp residues in this calculation (Table 2 and Figure S3). The thermal stability of the possible isoforms of zebrafish type I collagen was predicted to be higher in the order of (α1)_3_, [(α1)_2_α3], [(α1)_2_α2], and [α1α2α3]. The effect of chain stagger was too little to affect the order of the thermal stability of the isoforms. The simulation results suggest that isoforms with more α1-chains have relatively higher thermal stability. Data shown in Table 1 reveal that the sums of ratios of relatively stable isoforms ((α1)_3_ + [(α1)_2_α3] and (α1)_3_ + [(α1)_2_α2]) increased at 33 °C, whereas the relatively unstable [α1α2α3] ratio decreased. Thus, changes in α-chain composition may contribute to the higher thermal stability of secreted collagen at 33 °C.

## 3. Discussion

Because the enzymes involved in procollagen post-translational modifications recognize only the single-stranded portions as substrate [12], the modifications should last until triple helix-formation is completed in the ER. The amount of 4-Hyp in collagen was reported to increase in a temperature-dependent manner when full-length unmodified-polypeptide chains were treated with prolyl 4-hydroxylase [34,35,36]. Furthermore, 4-Hyp stabilizes the triple-helical structure significantly [2]; 3-hydroxylation of Pro may also stabilize it [6]. Modifications of Lys side chains affect the thermal stability of the triple helix [7,8]. The secretion of procollagen involves TANGO1; *Tango1*-null mice show abnormal bone formation due to delayed collagen secretion [37]. TANGO1 is involved in the formation of large COPII vesicles [38,39] and tunnels from the ER to the Golgi [40]. TANGO1 probably contributes to the selective secretion of procollagen forming the triple helix by interacting with HSP47, which binds to the triple-helical structure of procollagen [41]. By contrast, misfolded procollagen is recognized by calnexin in the ER and degraded through autophagy [13]. These findings suggest that procollagen is stabilized in the ER lumen by either extensive post-translational modifications or assistance by HSP47 until the formation of the triple helix is completed. Only procollagen molecules with the triple-helical structure are secreted to the extracellular space via the Golgi. In this study, we hypothesized a mechanism that provides sufficient thermal stability based on environmental temperature and tested this hypothesis.

Quantification of post-translationally modified amino-acid residues in zebrafish type I collagen secreted at different temperatures showed a temperature-dependent increase in 4-Hyp levels. However, the rate of the change was low (Figure 2), probably because the Pro residues at position Y receive 4-hydroxylation nearly quantitatively even at 18 °C. The amount of total Hyl also increased with increasing culture temperature. An increase in the amount of 3-Hyp and glycosylated Hyl was observed only at 33 °C, indicating a delay in triple helix-formation in the ER. The temperature-dependent changes in the ratios of such minor modifications appeared more significantly than major modifications, such as prolyl 4-hydroxylation, probably because more unmodified substrate sequences remained in α-chains. Quantification of α-chains of type I collagen secreted at different temperatures revealed that the ratio of α1-chain was higher, and the ratios of α2 and α3-chains were lower only at 33 °C (Figure 4). Simulation analysis using the algorithm suggested that the change in α-chain composition at 33 °C increases the thermal stability of collagen (Table 1 and Table 2). Although the simulation cannot predict the actual melting temperature of the collagen triple helix, it allowed a relative comparison of thermal stability among the collagen isoforms. The melting temperatures of the collagen triple helix were almost constant between culture temperatures 18 and 28 °C, whereas only collagen secreted at 33 °C showed a higher melting temperature (Figure 5). This is probably due to factors including an increase in prolyl 3-hydroxylation (Figure 2) and changes in the α-chain composition of type I collagen (Figure 4). An increase of Hyl glycosylation (Figure 1) may also affect thermal stability. The significant difference in the character of zebrafish type I collagen secreted at 33 °C would be a consequence of the quality control mechanism ensuring triple-helical conformation. However, we cannot exclude the possibility of secretion of unfolded collagen that could have been degraded with pepsin treatment at 4 °C during sample preparation.

**Figure 6:**
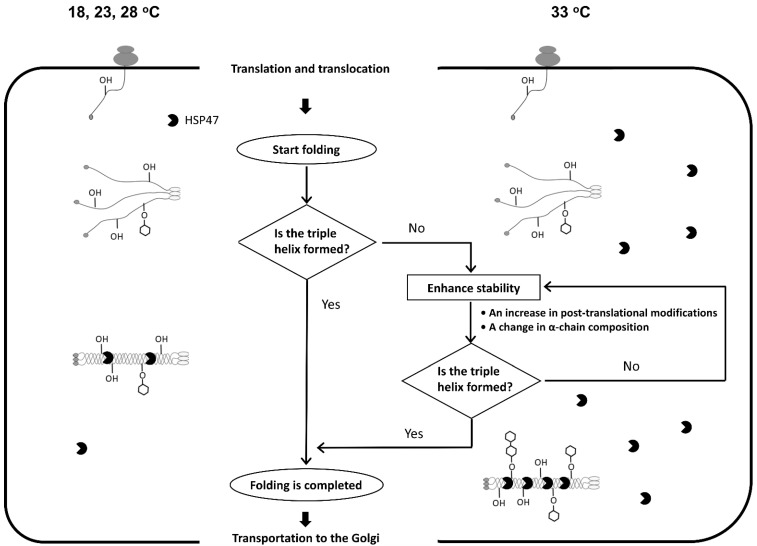
A possible mechanism for tuning triple-helix stability according to the environmental temperature. When ZF4 cells were cultured below 28 °C, the thermal stability of the procollagen triple helix is almost constant because of the contribution of co-translational modifications. At a higher temperature of 33 °C, the additional mechanism to ensure thermal stability of procollagen triple helix is executed.

Post-translational modifications of procollagen α-chains start immediately after translocating to the ER lumen [12]. Co-translational modifications and interaction with HSP47 provide sufficient stability to the triple helix to be folded below 28 °C (Figure 6). Increased post-translational modifications, altered α-chain composition, and the resulting increase in thermal stability of collagen secreted by ZF4 cells at 33 °C are similar to collagen abnormalities observed in *hsp47*-null mouse fibroblasts [18]. They may be due to the destabilization of the procollagen triple helix in the ER. Although ZF4 cells induce the expression of HSP47 at 33 °C, it may have been insufficient to cope with the destabilization of the procollagen triple-helical structure. Therefore, procollagen has acquired higher thermal stability by further post-translational modifications and changes in α-chain composition (Figure 6). Consistent with the results of this study, it has been reported that human skin fibroblasts also secrete over-modified collagen when cultured at a high temperature of 40.5 °C [42].

In vivo, temperature differences may lead to collagen production with different properties. Collagen in muscle tissue was reported to show higher thermal stability than collagen in skin tissue [43,44,45]. In addition, the amount of 4-Hyp and Hyl in collagen could also vary depending on the tissue [46]. These results indicate that differences in temperature in tissues may cause these differences. Core body temperature is higher than peripheral body temperature in homeotherms, and this temperature difference can also affect the properties of collagen. The melting temperature of collagen in human bone is higher than that of the skin, and an increase of post-translational modifications in bone collagen has been observed [47].

Mammalian type I collagen molecule is a [(α1)_2_α2] heterotrimer. However, the presence of (α1)_3_ homotrimer in fetal [48] and cancer [49,50,51,52,53] tissues has been elucidated. Cancer cells can reportedly migrate along the collagenase-resistant homotrimers as a scaffold [49]. Consistent with results from the simulation of thermal stability comparison among different zebrafish type I collagen molecules (Table 2), the melting temperature of mammalian (α1)_3_ homotrimers is higher than that of [(α1)_2_α2] heterotrimers [54,55]. The temperature of breast cancer tissue was reported to be higher than that of surrounding normal tissues, with a difference of up to 3.5 °C [56,57]. Our findings suggest that the secretion of the relatively higher thermally stable isoforms, with more α1-chains ((α1)_3_, [(α1)_2_α3], [(α1)_2_α2]), increased at a higher temperature of 33 °C (Table 1 and Table 2). High temperatures may contribute to the secretion of (α1)_3_ homotrimers that have higher thermal stability than [(α1)_2_α2] heterotrimers in mammalian tissues. We showed that the thermal stability of the collagen triple helix could be tuned depending on the environmental temperature by altering the amount of post-translational modifications and α-chain compositions. It is tempting to speculate that collagen with different properties produced through this mechanism has different roles in vivo.

## 4. Materials and Methods

### 4.1. Cell Culture

ZF4 cells were purchased from ATCC (CRL-2050). The cells were cultured in a 1:1 mixture of Dulbecco’s Modified Eagle’s Medium and Ham’s F-12 (DMEM/Ham’s F12, Wako Pure Chemical Industries, Osaka, Japan) supplemented with 10% fetal bovine serum (FBS), 100 U/mL penicillin, and 100 µg/mL streptomycin (Sigma-Aldrich, St. Louis, MO, USA) at 28 °C in an atmosphere of 5% CO_2_.

For all experiments, the cells were maintained at 18, 23, 28, or 33 °C for one day for acclimation after they became confluent. The medium was replaced with DMEM/Ham’s F12 containing 200 µM L-ascorbic acid phosphate magnesium salt *n*-hydrate (Wako Pure Chemical Industries, Osaka, Japan), 2% FBS, 100 U/mL penicillin, and 100 µg/mL streptomycin. The cells were maintained at 18, 23, 28, or 33 °C in a 5% CO_2_ atmosphere for 3 days and used for the experiments.

### 4.2. SDS-PAGE Analysis of Pepsin-Treated Collagen Samples

Culture media from ZF4 cells cultured at different temperatures for 3 days was centrifuged (2290× *g*) at 4 °C for 15 min, and the supernatant was treated with 100 µg/mL pepsin (Sigma-Aldrich, St. Louis, MO, USA) in 0.1 N HCl at 4 °C for 16 h. Collagen samples were isolated by salting out (1 M NaCl/0.1 N HCl) at 4 °C for 3 h. The precipitates obtained through centrifugation (4 °C, 20,900× *g*, 15 min) were dissolved in SDS-PAGE sample buffer (50 mM Tris-HCl [pH 6.7], 10% glycerol, 2% SDS, and 0.002% bromophenol blue) and heated at 95 °C for 5 min. SDS-PAGE was performed on a 5% gel under nonreducing conditions, and the protein bands were visualized with CBB.

### 4.3. Western Blotting Analysis of Culture Media Samples and Pull-Down Samples

After the culture media was collected from ZF4 cells cultured at different temperatures for 3 days, the cells were washed with phosphate-buffered saline (PBS) and treated with lysis buffer (1% NP-40, 150 mM NaCl, 5.0 mM EDTA, 50 mM Tris-HCl (pH 8.0), 2.0 mM *N*-ethylmaleimide, 1.0 mM phenylmethylsulfonyl fluoride, 1 µg/mL leupeptin, and 1 µg/mL pepstatin A) on ice for 15 min. After centrifugation (20,600× *g*) at 4 °C for 15 min, soluble protein in the supernatant was quantified using the Bradford method. For concentrating HSP47, 500 μL of the lysate (protein concentration: 5 mg/mL) was mixed with 100 μL-bed of type I collagen (AteloCell I-AC30, Koken, Tokyo, Japan), coupled or mock-coupled with CNBr-activated Sepharose 4B (GE Healthcare, Piscataway, NJ, USA) beads. Samples were rotated at 4 °C for 2 h and washed with 0.4 M NaCl/PBS. Then, the protein bound to the beads was eluted by adding 100 μL of 2 × SDS sample buffer, followed by heating at 95 °C for 5 min. To prepare the lysate and media samples, 5 × SDS sample buffer was added to the lysate and culture medium and heated at 95 °C for 5 min.

For western blot analysis of media samples, the amount of medium per unit protein extracted from the cell layer was calculated and loaded onto the gel. SDS-PAGE was performed using a 5% gel for the media samples in the presence of 91 mM 1,4-dithiothreitol (DTT) or 10% gel for the pull-down samples. Proteins were transferred to nitrocellulose membranes. The membranes were blocked with 5% skim milk/Tris-buffered saline (TBS; 50 mM Tris-HCl pH 7.4, 150 mM NaCl) for 1 h and washed with TBS. The membranes were treated with the primary antibody or 3 μg/mL of biotin-labeled soCMP6-7(Glu)2 in 2% skim milk/TBS to detect HSP47 or collagen [24], respectively. Then, the membranes were washed with TBS, treated with the secondary antibody or streptavidin-alkaline phosphatase (Promega, Madison, WI, USA, 1:1000 dilution) in 2% skim milk/TBS, respectively, and washed with TBS containing 0.1% Tween-20. HSP47 bands were detected using the CCD imager LAS-3000 (Fujifilm, Tokyo, Japan) with Pierce™ ECL western blotting substrate kit (Thermo Fisher Scientific, Waltham, MA, USA). Collagen bands were visualized using the alkaline phosphatase conjugate substrate kit (Bio-Rad Laboratories, Hercules, CA, USA).

The primary antibodies used included the anti-SERPINH1 rabbit polyclonal antibody (MBS835022, MyBioSource, San Diego, CA, USA, 1:250 dilution) and anti-β-actin monoclonal antibody (A5316, Sigma-Aldrich, St. Louis, MO, USA, 1:2000 dilution); the secondary antibodies were goat anti-rabbit IgG-HRP conjugate (7074S, Cell Signaling Technology, Danvers, MA, USA, 1:2000 dilution), and goat anti-mouse IgG-HRP conjugate (W402B, Promega, Madison, WI, USA, 1:5000 dilution), respectively.

### 4.4. Synthesis of Stable Isotope-Labeled Peptides

Stable isotope-labeled marker peptides corresponding to tryptic marker peptides for quantifying zebrafish α1(I), α2(I), and α3(I)-chains were prepared by 9-fluorenylmethoxycarbonyl (Fmoc) solid-phase peptide synthesis on Wang resin using Fmoc-^15^N-Gly-OH (Sigma-Aldrich, St. Louis, MO, USA). The peptide resin was treated with trifluoroacetic acid/H_2_O/*m*-cresol/thioanisole/1,2-ethanedithiol (82.5/5/5/5/2.5, *v*/*v*) for 2 or 4 h at about 25 °C. The deprotected peptides were purified through RP-HPLC on a Cosmosil 5C18-AR-II column (6.0 mm × 250 mm, Nacalai Tesque, Kyoto, Japan) with CH_3_CN in water, both containing 0.05% (*v*/*v*) TFA. The desired peptides were analyzed using RP-HPLC on a Cosmosil 5C18-AR-II column (4.6 mm × 250 mm, Nacalai Tesque, Kyoto, Japan) with a linear gradient of CH_3_CN in H_2_O, both containing 0.05% (*v*/*v*) TFA. Mass spectrometric analysis was performed with Autoflex III MALDI-TOF MS (Bruker Daltonics, Bremen, Germany). To determine the absolute amount of these peptides, amino-acid analysis was performed using an L-8900 amino-acid analyzer (Hitachi, Tokyo, Japan) after acid hydrolysis with 6 N HCl/1% phenol at 110 °C for 20 h in the gas phase under N_2_.

### 4.5. Quantification of Type I Collagen α-Chains by LC-MS

The culture media of ZF4 cells cultured for 3 days was centrifuged (2290× *g*, 4 °C, 15 min), and the supernatant was collected. The supernatant was treated with pepsin at 4 °C in 0.1 N HCl after adding SI-collagen, and collagen was isolated through salt precipitation. The collagen samples were heat-denatured at 60 °C for 30 min and digested with sequencing grade modified trypsin (Promega, Madison, WI, USA) at 37 °C for 16 h in 100 mM Tris-HCl/1 mM CaCl_2_ (pH 7.6), after adding the stable isotope-labeled peptides as internal standards. We selected two tryptic marker peptides for each α-chain (α1(I) 421-434, α1(I) 688-704, α2(I) 351-360, α2(I) 502-519, α3(I) 295-309, and α3(I) 421-434). The marker peptides were analyzed by LC-MS in multiple reaction monitoring (MRM) mode on a 3200 QTRAP hybrid triple quadrupole (QqQ)/linear ion trap mass spectrometer (AB Sciex, Foster City, CA, USA) coupled to an Agilent 1200 Series HPLC system (Agilent Technologies, Palo Alto, CA, USA) using the BIOshell A160 Peptide C18 HPLC column (5 μm particle size, L× I.D. 150 mm × 2.1 mm; Supelco, Bellefonte, PA, USA) to quantify each α-chain based on the peak area ratio relative to the corresponding internal standard peptides as described [18]. Stable isotopically heavy human type I collagen marker peptides from SI-collagen [27] were also analyzed to correct sampling errors. After quantifying the 1% NP-40 soluble protein in the cells as shown in Section 4.3, the amount of secreted type I collagen α-chains per amount of protein in the cells was calculated (Table S3).

### 4.6. Quantification of Total Post-Translational Modifications in Collagen by LC-MS

Total levels of post-translational modifications in collagen were estimated by LC-MS as described [18]. Briefly, collagen samples purified from the culture media of ZF4 cells were subjected to SDS-PAGE (5% gel) under nonreducing conditions, and the gel was electroblotted onto a PVDF membrane. The membrane was stained with CBB, and the bands of α1(I) + α3(I) and α2(I)-chains were cut out from the membrane. SI-collagen was added as an internal standard, and the excised bands were subjected to acid hydrolysis (6 N HCl/1% phenol, 110 °C for 20 h in the gas phase under N_2_). Pro, 3-Hyp, 4-Hyp, Lys, and total Hyl (Hyl + glycosylated Hyl) were quantified by LC-QqQ-MS in MRM mode following chromatographic separation using the ZIC-HILIC column (3.5 μm particle size, L × I.D. 150 mm × 2.1 mm; Merck Millipore, Billerica, MA, USA) [18].

### 4.7. Site-Specific Characterization of Prolyl 3-Hydroxylation in Zebrafish Type I Collagen by LC-MS

The purified collagen samples were digested with trypsin as described above to evaluate the relative abundance of prolyl 3-hydroxylation at specific sites in zebrafish type I collagen by LC-MS. The tryptic digests were analyzed by LC-MS on the maXis II quadrupole time-of-flight mass spectrometer (Bruker Daltonics, Bremen, Germany) coupled to the Shimadzu Prominence UFLC-XR system (Shimadzu, Kyoto, Japan) using the Ascentis Express C18 HPLC column (5 μm particle size, L × I.D. 150 mm × 2.1 mm; Supelco, Bellefonte, PA, USA). Site occupancy of prolyl 3-hydroxylation at respective modification sites was semi-quantitatively estimated by the relative peak area ratio of monoisotopic extracted ion chromatograms for each 3-Hyp variant of tryptic peptides containing the modification sites described previously [58].

### 4.8. Calculation of the Amount Ratios of Possible Trimers in Type I Collagen

The amount ratios of α1, α2, and α3-chains to total α-chains (α1 + α2 + α3) of type I collagen are described as r_α1_, r_α2_, and r_α3_, respectively. The amount ratios of (α1)_3_, [(α1)_2_α3], [(α1)_2_α2], and [α1α2α3] to total trimers ((α1)_3_ + [(α1)_2_α3] + [(α1)_2_α2] + [α1α2α3]) of type I collagen are described as R_(__α1)3_, R_[(α1)2α3]_, R_[(α1)2α2]_, and R_[α1α2α3]_, respectively. r_α1_, r_α2_, and r_α3_ are formulated from R_(__α1)3_, R_[(α1)2α3]_, R_[(α1)2α2]_, and R_[α1α2α3]_.

r_α1_ = (3R_(__α1)3_ + 2R_[(α1)2α3]_ + 2R_[(α1)2α2]_ + R_[α1α2α3]_)/3 (1)

r_α2_ = (R_[(α1)2α2]_ + R_[α1α2α3]_)/3 (2)

r_α3_ = (R_[(α1)2α3]_ + R_[α1α2α3]_)/3 (3)

To calculate the ratios of trimers using the observed data, r_α1_, r_α2_, and r_α3_, shown in Figure 4 and Table S4, Equations (1)–(3) were organized as Equations (4)–(6).

R_(__α1)3_ = r_α1_ − 2r_α2_ + r_α3_ − R_[(α1)2α3]_ (4)

R_[(α1)2α2]_ = 3(r_α2_ − r_α3_) + R_[(α1)2α3]_ (5)

R_[α1α2α3]_ = 3r_α3_ − R_[(α1)2α3]_ (6)

The amount ratios of individual trimers were calculated from Equations (4)–(6), and the results are shown in Table 1. R _[(α1)2α3]_ at 18, 23, 28, and 33 °C was given as variables x, y, z, and w, respectively. As all the trimer ratios must be positive values, the variable x is greater than or equal to 9.0 so that R_[(α1)2α2]_ at 18 °C is 0 or more. The other variables y, z, and w are all greater than or equal to 0 so that R_[(α1)2α3]_ at 23, 28, and 33 °C are 0 or more. In addition, x ≤ 15.1, y ≤ 9.4, z ≤ 11.1, and w ≤ 29.9 are satisfied so that R_(__α1)3_ is 0 or more.

### 4.9. Measurement of the Melting Temperature of Collagen

The collagen samples purified from the culture media of ZF4 cells by pepsin digestion and salt precipitation were dissolved in 10 mM acetic acid, and CD signals at 221 nm were measured with the J-820 CD spectropolarimeter (Jasco, Tokyo, Japan) equipped with a Peltier thermal controller while heating (0.25 °C/min). CD signals at 221 nm were normalized to the fraction folded. Tm values are temperatures at which fraction folded is 0.5.

### 4.10. Prediction of Relative Thermal Stability of Possible Isoforms of Zebrafish Type I Collagen

The amino-acid sequences of the α1, α2, and α3-chains of zebrafish type I collagen were obtained from Uniprot database (https://www.uniprot.org/, accessed on 5 January 2022; *D. rerio* α1(I): NP_954684.1, α2(I): NP_892013.2, α3(I): NP_958886.1). All the Pro at position Y in the triple-helical region (Gly-X-Y) consisting of 1014 amino-acid residues were converted to 4-Hyp. The converted sequences of the triple-helical regions were divided into 334 sequences of 15 amino-acid residue fragments by shifting three residues from each α-chain. The relative stability of 334 sites was predicted as the sequences attached five repeats of Gly-Pro-4-Hyp triplets at both N- and C-terminus for the 10 possible isoforms of zebrafish type I collagen by SCEPTTr (Figure S3) [31]. Chain stagger was adopted with one-residue offset. The virtual relative thermal stability of each isoform was calculated as the average of predicted relative stability for 334 peptides of 45 amino-acid residues.

### 4.11. Statistical Analysis

The results are presented as means ± SD. Differences of means were assessed by one-way ANOVA and Tukey’s multiple comparison test using a GraphPad Prism software version 7.04 (GraphPad Software, La Jolla, CA, USA).

## 5. Conclusions

In general, the thermal stability of a protein conformation is determined a priori by its primary structure. In this study, by using poikilotherm cells, we elucidated that the thermal stability of the collagen triple helix was tuned according to the environmental temperature. The secreted collagen acquires higher thermal stability by a change in α-chain composition and an increase in post-translational modifications through an ad hoc mechanism at a high temperature above the threshold.

## Figures and Tables

**Table 1:** Trimer ratios in type I collagen secreted from ZF4 cells.

Culture Temperature	(α1)_3_	[(α1)_2_α3]	[(α1)_2_α2]	[α1α2α3]	(α1)_3_ + [(α1)_2_α3]	(α1)_3_ + [(α1)_2_α2]
	Average (SD) (%)	Average (SD) (%)	Average (SD) (%)	Average (SD) (%)	Average (SD) (%)	Average (SD) (%)
18 °C	15.1 (3.1) − x *max: 6.1 (x = 9.0)* *min: 0 (x = 15.1)*	x *max: 15.1 (x = 15.1)* *min: 9.0 (x = 9.0)*	−9.0 (7.6) + x *max: 6.1 (x = 15.1)* *min: 0 (x = 9.0)*	93.9 (5.4) − x *max: 84.6 (x = 9.0)* *min: 78.5 (x = 15.1)*	15.1 (3.1)	6.1 (5.4)
23 °C	9.4 (2.2) − y *max: 9.4 (y = 0)* *min: 0 (y = 9.4)*	y *max: 9.4 (y = 9.4)* *min: 0 (y = 0)*	3.7 (4.5) + y *max: 13.1 (y = 9.4)* *min: 3.7 (y = 0)*	86.9 (3.3) − y *max: 86.9 (y = 0)* *min: 77.8 (y = 9.1)*	9.4 (2.2)	13.1 (3.3)
28 °C	11.1 (3.5) − z *max: 11.1 (z = 0)* *min: 0 (z = 11.1)*	z *max: 11.1 (z = 11.1)* *min: 0 (z = 0)*	2.4 (11.2) + z *max: 13.5 (z = 11.1)* *min: 2.4 (z = 0)*	86.4 (7.8) − z *max: 86.4 (z = 0)* *min: 75.3 (z = 11.1)*	11.1 (3.5)	13.6 (7.8)
33 °C	29.9 (8.2) − w *max: 29.9 (w = 0)* *min: 0 (w = 29.9)*	w *max: 29.9 (w = 29.9)* *min: 0 (w = 0)*	4.9 (15.4) + w *max: 34.8 (w = 29.9)* *min: 4.9 (w = 0)*	65.2 (7.3) − w *max: 65.2 (w = 0)* *min: 35.3 (w = 29.9)*	29.9 (8.2)	34.8 (7.3)

9.0 ≤ x ≤ 15.1, 0 ≤ y ≤ 9.4, 0 ≤ z ≤ 11.1, 0 ≤ w ≤ 29.9. *n* = 3.

**Table 2:** Simulation of the relative thermal stability of zebrafish type I collagen with different chain compositions.

α-Chain Composition	Chain Stagger	Virtual Relative Thermal Stability (°C)
(α1)_3_	α1α1α1	40.7
[(α1)_2_α3]	α1α1α3	39.9
	α3α1α1	39.7
	α1α3α1	39.3
[(α1)_2_α2]	α2α1α1	39.0
	α1α1α2	38.8
[α1α2α3]	α2α3α1	38.0
	α2α1α3	37.9
	α1α3α2	37.7
	α3α1α2	37.5

## Data Availability

The data presented in this study are available in the article or Supplementary Materials.

## Supplementary Materials

The following supporting information can be downloaded at: https://www.mdpi.com/article/10.3390/ijms23042040/s1.
