# Supplementary material for: The Thermal Stability of the Collagen Triple Helix Is Tuned According to the Environmental Temperature
Source: Int J Mol Sci. 2022 Feb 12;23(4):2040. doi: 10.3390/ijms23042040 (PMC8877210; doi:10.3390/ijms23042040)
Supplement: Supplementary file 1 [file ijms-23-02040-s001.zip › ijms-1566872-supplementary.pdf]

Supplementary materials

The thermal stability of the collagen triple helix is tuned according to the environmental temperature

Kazunori K Fujii[1], Yuki Taga[2], Yusuke K Takagi[1], Ryo Masuda[3], Shunji Hattori[2], Takaki Koide[1,3,*]

[1]Department of Chemistry and Biochemistry, School of Advanced Science and Engineering, Waseda University, Shinjuku, Tokyo 169-8555, Japan; k.fujii@ruri.waseda.jp (K.K.F.); takagi.yt@moegi.waseda.jp (Y.K.T.)

[2]Nippi Research Institute of Biomatrix, 520-11 Kuwabara, Toride, Ibaraki 302-0017, Japan; y-taga@nippi-inc.co.jp (Y.T.); shunhatt@nippi-inc.co.jp (S.H.)

[3]Waseda Research Institute for Science and Engineering, Waseda University, Shinjuku, Tokyo 169-8555, Japan; r.masuda@aoni.waseda.jp (R.M.)

*Correspondence: koi@waseda.jp; Tel.: +81-3-5286-2569

Quantification of the total post-translational modifications of type I collagen secreted from ZF4 cells

Pro, 3-Hyp, 4-Hyp, Lys, and total Hyl (Hyl + glycosylated Hyl) in α1 + α3 and α2-chains of type I collagen secreted from ZF4 cells were quantified by LC-MS after acid hydrolysis. The corresponding stable isotope-labeled analytes derived from SI-collagen were used as internal standards. The numbers per 1000 residues in the triple-helical region were calculated (Table S1).

Table S1. Total post-translational modifications in type I collagen secreted from ZF4 cells (residues/1000 residues).

		Pro		3-Hyp		4-Hyp		Lys		Hyl (total)	
		Average	SD	Average	SD	Average	SD	Average	SD	Average	SD
α1(I) + α3(I)	18 °C	126.8	6.7	4.9	0.1	121.7	2.7	33.1	1.3	11.4	0.8
	23 °C	126.5	16.3	3.5	0.4	119.1	13.2	29.1	2.0	12.6	0.7
	28 °C	132.7	8.9	4.5	0.6	134.2	13.0	25.9	1.6	20.4	2.2
	33 °C	109.2	7.8	6.2	0.4	117.7	7.2	25.2	0.6	24.3	1.6
α2(I)	18 °C	124.6	14.0	1.3	0.2	84.8	12.8	31.3	1.6	11.0	1.4
	23 °C	144.0	12.0	1.2	0.1	104.8	7.7	29.7	2.5	16.8	2.5
	28 °C	131.5	15.4	1.3	0.2	99.0	19.0	29.3	7.0	18.2	2.4
	33 °C	137.6	12.3	3.4	0.9	113.1	7.9	22.7	1.1	23.2	4.9

$n = 3$

Calculation of the maximum ratios of residues converted to 4-Hyp or Hyl

The ratio of possible residues converted to 4-Hyp was calculated as the number of Pro at position Y divided by that of total Pro (Table S2a). The ratio of possible residues converted to Hyl was calculated as the number of Lys at position Y divided by that of total Lys (Table S2b). Their numbers in the triple-helical region (1014 amino acid residues) were counted from zebrafish α1(I) (NCBI accession number: NP_954684.1), α2(I) (NCBI accession number: NP_892013.2), and α3(I) (NCBI accession number: NP_958886.1).

Table S2. The numbers of Pro and Lys placed at positions X and Y in zebrafish type I collagen triple helix.

(a)

	**Pro in X position (x)**	**Pro in Y position (y)**	**putative 4-Hyp ratio (y/(x + y))**
**α1**	101	102	50.2% (102/203)
**α2**	111	85	43.4% (85/196)
**α3**	105	103	49.5% (103/208)

(b)

	**Lys in X position (x)**	**Lys in Y position (y)**	**putative Hyl ratio (y/(x + y))**
**α1**	12	23	65.7% (23/35)
**α2**	9	22	71.0% (22/31)
**α3**	12	20	62.5% (20/32)

(a)

				707									716			719						
**zebrafish**	**α1[705–725]**	V	G	**P**	O	G	P	S	G	N	S	G	**P**	O	G	**P**	O	G	P	A	G	K
	**α3[705–725]**	V	G	**P**	O	G	P	S	G	A	S	G	**P**	O	G	P	T	G	P	A	G	K
**rat**	**α1[705–725]**	V	G	P	O	G	P	S	G	N	A	G	P	O	G	P	O	G	P	V	G	K

				707									716			719						
**zebrafish**	**α2[705–725]**	V	G	**P**	O	G	P	A	G	I	V	G	P	A	G	L	T	G	P	A	G	K
**rat**	**α2[705–725]**	T	G	P	O	G	P	S	G	I	T	G	P	O	G	P	O	G	A	A	G	K

(b)

													986				
**zebrafish**	**α1[975–990]**	D	G	M	S	G	L	O	G	P	I	G	**P**	O	G	P	R
	**α3[975–990]**	D	G	M	N	G	V	O	G	P	V	G	**P**	O	G	P	R
**rat**	**α1[975–990]**	D	G	L	N	G	L	O	G	P	I	G	P	O	G	P	R

													986				
**zebrafish**	**α2[975–990]**	D	G	S	N	G	M	O	G	A	I	G	**P**	O	G	H	R
**rat**	**α2[975–990]**	D	G	R	S	G	H	O	G	P	V	G	P	A	G	V	R

Figure S1. Comparison of amino acid sequences around the possible prolyl 3-hydroxylation sites in type I collagen.

Comparison of tryptic peptides (a) [705–725] and (b) [975–990] of zebrafish type I collagen with their corresponding amino acid sequences in rat type I collagen. Pro residues with 3-hydroxylation, in this study, are shown in bold. Pro residues reported as possible 3-hydroxylation sites are underlined [1].

Quantification of the amount ratios of possible trimers in type I collagen secreted from ZF4 cells

The results of quantification for α-chains of type I collagen secreted from ZF4 cells are shown in Table S3. The values of amount ratios of type I collagen α-chains were calculated based on the quantitative values (Figure 4 and Table S4). The values were assigned $r_{\alpha1}$, $r_{\alpha2}$, or $r_{\alpha3}$ in Section 4.8 to estimate $R_{(\alpha1)3}$, $R_{[(\alpha1)2\alpha3]}$, $R_{[(\alpha1)2\alpha2]}$, and $R_{[\alpha1\alpha2\alpha3]}$, respectively.

Table S3. The quantitative values of α-chains of type I collagen secreted from ZF4 cells.

	α1		α2		α3	
	Average (pmol/mg)	SD (pmol/mg)	Average (pmol/mg)	SD (pmol/mg)	Average (pmol/mg)	SD (pmol/mg)
**18 °C**	4.40	0.88	3.07	0.48	3.42	0.73
**23 °C**	10.71	0.78	7.93	0.69	7.59	0.33
**28 °C**	14.05	1.41	10.01	0.97	9.69	0.47
**33 °C**	8.66	2.63	3.76	1.49	3.38	0.85

n = 3

Table S4. The values of the amount ratios of α-chains in type I collagen secreted from ZF4 cells.

	α1 ratio ($r_{\alpha1}$)		α2 ratio ($r_{\alpha2}$)		α3 ratio ($r_{\alpha3}$)	
	Average (%)	SD (%)	Average (%)	SD (%)	Average (%)	SD (%)
**18 °C**	40.4	1.4	28.3	1.0	31.3	1.8
**23 °C**	40.8	1.1	30.2	0.7	29.0	1.1
**28 °C**	41.6	1.5	29.6	1.2	28.8	2.6
**33 °C**	54.9	0.5	23.4	2.7	21.7	2.4

n = 3

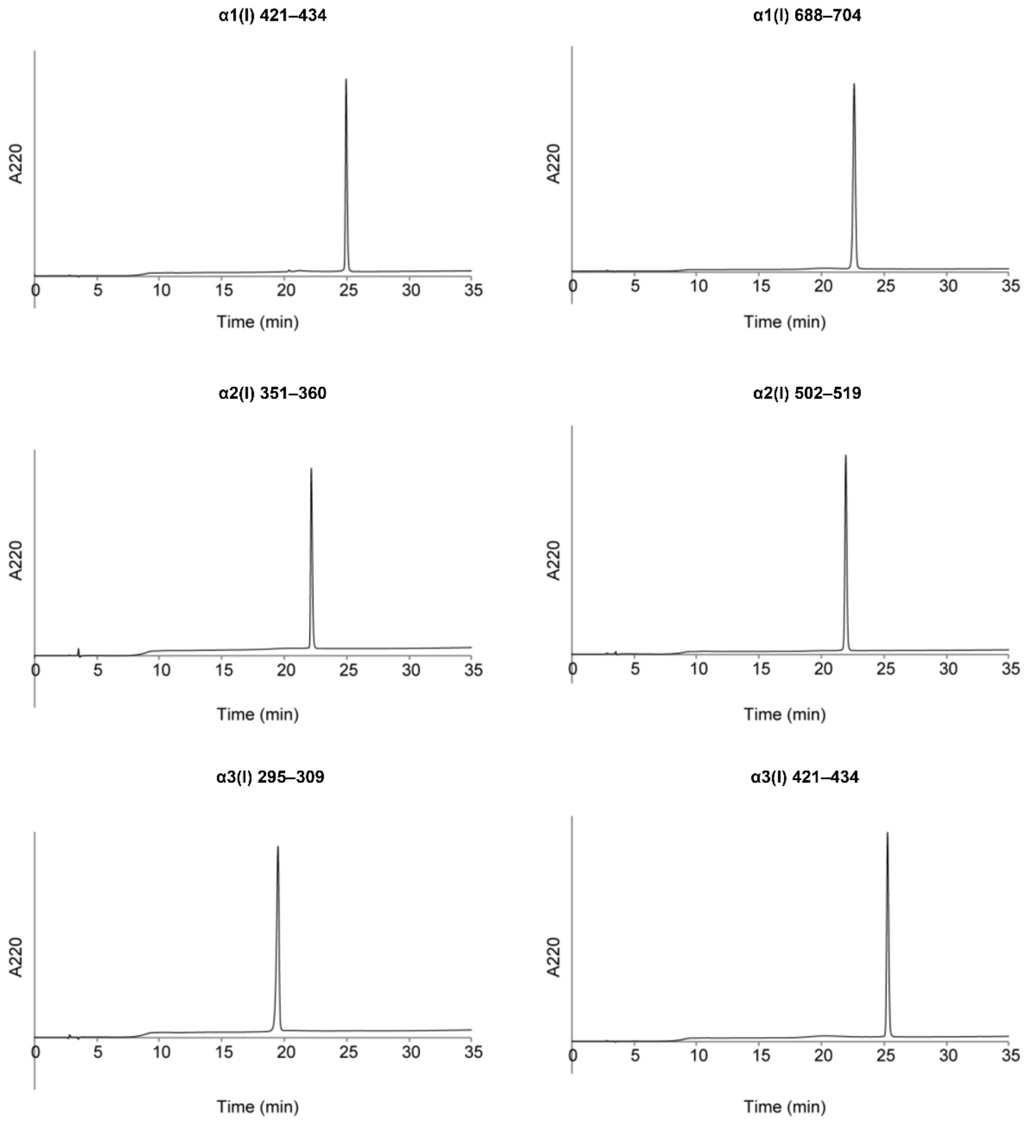

Figure S2. HPLC profiles of synthetic peptides.

The peptides were analyzed by RP-HPLC using the COSMOSIL 5C_{18}-AR-II column (4.6 mm × 250 mm, Nacalai Tesque). HPLC gradient: 0%–30% CH_3CN in 0.05% TFA over 30 min and 90% CH_3CN in 0.05% TFA over 5 min. Detection: 220 nm. Flow rate: 1 mL/min.

Table S5. Mass spectrometric analysis of the synthetic peptides.

peptide	sequence	found	calculated monoisotopic mass [M + H]
**α1(I) 421–434**	**GVMGAIGATGAOGK**	1207.793	1207.605
**α1(I) 688–704**	**GAAGPOGATGFOGAAGR**	1449.541	1449.680
**α2(I) 351–360**	**AGEAGLVGAR**	903.700	903.481
**α2(I) 502–519**	**GNOGPAGAAGAQGPIGAR**	1541.095	1540.755
**α3(I) 295–309**	**GEOGAAGPVGPOGAR**	1326.983	1326.635
**α3(I) 421–434**	**GLAGPTGPLGAOGK**	1213.869	1213.649

O: 4-hydroxyproline.

All Gly are ^{15}N-Gly.

(α1)$_3$

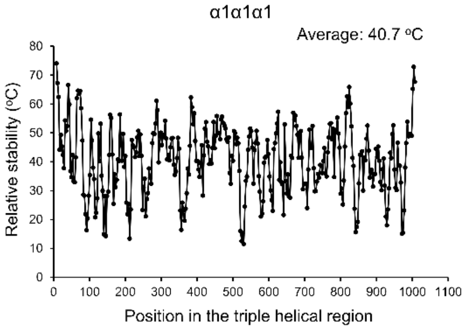

[(α1)$_2$α3]

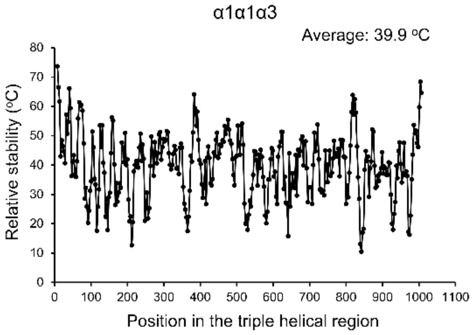

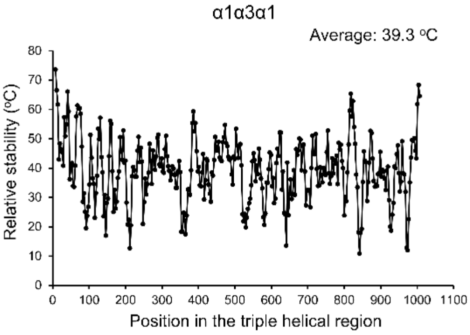

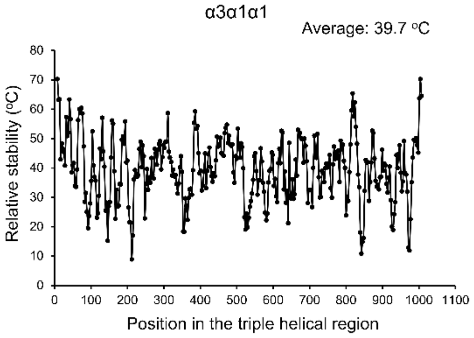

Figure S3. Virtual relative thermal stability of each possible isoform of zebrafish type I collagen.

The relative thermal stability of 334 fragments of possible isoforms of zebrafish type I collagen was predicted by SCEPTTr as described in Section 4.10 [2]. Each isoform's virtual relative thermal stability was calculated as the average of the predicted melting temperatures for 334 collagen-derived peptide fragments. It is plotted according to their corresponding position to collagen's triple-helical region consisting of 1014 amino acid residues.

[$(\alpha1)_2\alpha2$]

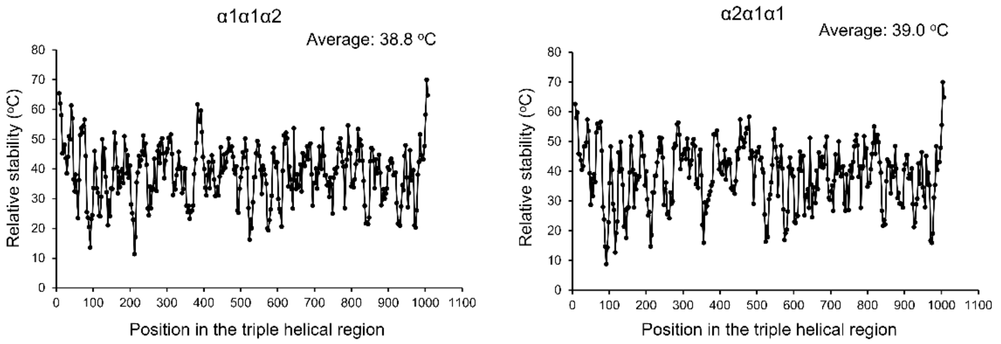

[$\alpha1\alpha2\alpha3$]

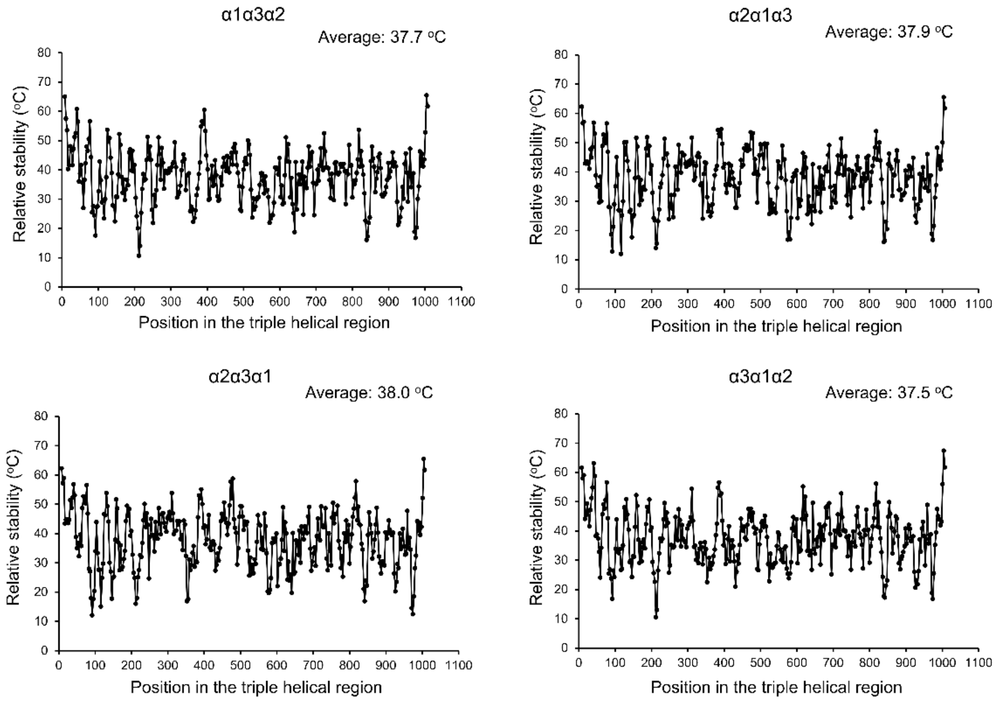

Figure S3. *Continued.*

References

[1] Taga, Y.; Kusubata, M.; Ogawa-Goto, K.; Hattori, S. Developmental Stage-Dependent Regulation of Prolyl 3-Hydroxylation in Tendon Type I Collagen. *J Biol Chem* **2016**, *291*, 837–847, doi:10.1074/jbc.M115.686105.

[2] Walker, D.R.; Hulgan, S.A.H.; Peterson, C.M.; Li, I.-C.; Gonzalez, K.J.; Hartgerink, J.D. Predicting the Stability of Homotrimeric and Heterotrimeric Collagen Helices. *Nat Chem* **2021***, 13*, 260–269, doi:10.1038/s41557-020-00626-6.
